# Exploring Multi-Pathology Brain Segmentation: From Volume-Based to Component-Based Deep Learning Analysis

**DOI:** 10.3390/jimaging11010006

**Published:** 2024-12-31

**Authors:** Ioannis Stathopoulos, Roman Stoklasa, Maria Anthi Kouri, Georgios Velonakis, Efstratios Karavasilis, Efstathios Efstathopoulos, Luigi Serio

**Affiliations:** 1 2nd Department of Radiology, Medical School, Attikon University Hospital, National and Kapodistrian University of Athens, 11527 Athens, Greece; ianstath@med.uoa.gr (I.S.); mariakouri90@gmail.com (M.A.K.); gvelonakis@med.uoa.gr (G.V.); 2 Technology Department, CERN, 1211 Geneva, Switzerland; stoklasa@fi.muni.cz (R.S.); luigi.serio@cern.ch (L.S.); 3 Centre for Biomedical Image Analysis, Faculty of Informatics, Masaryk University, 60200 Brno, Czech Republic; 4 Medical Physics Laboratory, School of Medicine, Democritus University of Thrace, 68100 Alexandroupolis, Greece; stratoskaravasilis@yahoo.gr

**Keywords:** deep learning, magnetic resonance imaging (MRI), AI algorithms, tumors, strokes, multiple sclerosis (MS), white matter hyperintensities (WMH), segmentation

## Abstract

Detection and segmentation of brain abnormalities using Magnetic Resonance Imaging (MRI) is an important task that, nowadays, the role of AI algorithms as supporting tools is well established both at the research and clinical-production level. While the performance of the state-of-the-art models is increasing, reaching radiologists and other experts’ accuracy levels in many cases, there is still a lot of research needed on the direction of in-depth and transparent evaluation of the correct results and failures, especially in relation to important aspects of the radiological practice: abnormality position, intensity level, and volume. In this work, we focus on the analysis of the segmentation results of a pre-trained U-net model trained and validated on brain MRI examinations containing four different pathologies: Tumors, Strokes, Multiple Sclerosis (MS), and White Matter Hyperintensities (WMH). We present the segmentation results for both the whole abnormal volume and for each abnormal component inside the examinations of the validation set. In the first case, a dice score coefficient (DSC), sensitivity, and precision of 0.76, 0.78, and 0.82, respectively, were found, while in the second case the model detected and segmented correct (True positives) the 48.8% (DSC ≥ 0.5) of abnormal components, partially correct the 27.1% (0.05 > DSC > 0.5), and missed (False Negatives) the 24.1%, while it produced 25.1% False Positives. Finally, we present an extended analysis between the True positives, False Negatives, and False positives versus their position inside the brain, their intensity at three MRI modalities (FLAIR, T2, and T1ce) and their volume.

## 1. Introduction

Brain abnormalities, ranging from tumors to strokes, multiple sclerosis (MS), and white matter hyperintensities (WMH), present formidable obstacles to neurological well-being. Tumors, whether benign or malignant, disrupt physiological brain function by exerting compressive forces on adjacent tissues, thereby impeding proper neurologic signaling. Strokes, precipitated by cerebral ischemia, engender severe consequences, including paralysis, cognitive deficits, or mortality. Multiple sclerosis, an autoimmune disorder, leads to the destruction of the protective myelin sheath covering nerve fibers, resulting in a wide array of neurological symptoms. White matter hyperintensities, often seen on MRI scans of older adults, are associated with small vessel disease and can indicate an increased risk of cognitive decline and stroke. Profound comprehension and effective management of these cerebral aberrations are imperative for safeguarding neurological integrity and enhancing patients’ quality of life [1,2,3,4].

To augment patient survival rates, early and accurate detection of brain abnormalities is imperative. Early diagnosis often facilitates efficacious and sustainable therapeutic interventions. However, detection and diagnosis of brain pathologies is not always trivial because of the complex size and structure of the human brain, while they highly depend on imaging equipment and the availability and expertise of the radiologist to identify, detect, and classify the pathology. Presently, Magnetic Resonance Imaging (MRI) plays an important role in the accurate diagnosis of several brain pathologies [5]. This noninvasive and radiation-free imaging modality enables the detection and characterization of brain tissue abnormalities. Tissue characterization is achieved due to the MRIs ability to combine different techniques to depict different tissues with different contrast [6].

Artificial intelligence (AI) offers promising avenues to cope with brain abnormalities by enhancing diagnosis, treatment, and management strategies. AI-powered algorithms can analyze vast amounts of medical data, including brain imaging scans such as MRIs and CT scans, to detect subtle abnormalities that might be missed by human observers [7,8]. This can lead to earlier and more accurate diagnoses, allowing for prompt intervention and treatment. Current research has demonstrated that convolutional neural networks (CNNs) can be proved highly efficient towards the detection and segmentation of brain abnormalities [9,10]. Most notably, the U-net model [11] has been gaining popularity in recent years thanks to its outstanding performances, especially for medical segmentation tasks. The U-net architecture, with its encoder-decoder structure, allows for the segmentation of fine details in the image and robust handling of partial volume effects, which are common in brain imaging. Pretrained U-net models have been widely used for segmentation of brain abnormalities in medical imaging [12]. These models are trained on large datasets of annotated images, such as Imagenet [13], and have achieved state-of-the-art performance in various segmentation tasks. By utilizing the transfer learning method and the pretrained weights, researchers can fine-tune the model for their specific task and achieve high accuracy with minimal training data [14].

The study by Zhang et al. [15] focused on brain tumor segmentation using several U-net variations, achieving a Dice Similarity Coefficient (DSC) score of 0.87, demonstrating superior segmentation performance compared to other methods. Gab Allah et al. [16] employed a custom edge-based U-net and a public dataset containing three tumor types, achieving an overall DSC of 88.8. Ferreira et al. [17] utilized a custom U-net with modern data augmentation techniques on a public tumor dataset, obtaining a DSC of 0.901. Huang et al. [18] applied a transfer-learning-based custom CNN and compared it with several U-net models for the segmentation of multiple sclerosis (MS), achieving a DSC of 0.66. Similarly, Pooya Ashtari et al. [19] implemented a custom U-net for MS segmentation on a public dataset, obtaining a DSC of 0.4. Yunzhe et al. [20] employed a multi-path CNN for lesion segmentation in both sub-acute and chronic strokes, achieving a DSC of 0.5 for smaller lesions. Jiong Wu et al. [21] developed a modified U-net with skip connections, achieving a DSC of 78.3 for White Matter Hyperintensities (WMH) segmentation. Guerrero et al. [22] used uResNet to segment both strokes and WMH, reporting DSC values ranging from 0.4 to 0.9, depending on the pathology.

Current research in deep learning for brain disease segmentation can be significantly advanced by focusing on the comprehensive evaluation of multi-pathology datasets and the performance of associated models. A holistic approach that leverages standard MRI modalities, homogenous datasets, and clinically oriented assessments is essential to pave the way for the clinical implementation of such techniques.

The primary goal of our research is to assess and evaluate the performance of a U-net deep learning model for segmenting an MRI-based, multimodal, and multi-pathology dataset while ensuring its relevance to everyday clinical practices. To achieve this, we have developed and integrated the following key components:

- A hospital-originated dataset incorporating three MRI modalities (FLAIR, T2, and T1ce) and four distinct pathologies: tumors, strokes, multiple sclerosis (MS), and white matter hyperintensities (WMH).
- A U-net model enhanced with transfer learning techniques.
- An evaluation framework for analyzing the entire pathology volume.
- A novel approach for evaluating each individual abnormal component by examining its spatial localization within the brain, intensity levels across modalities, and volume.

Our research aims to achieve a dual objective:

1. Identify the strengths and limitations of a modern U-net deep learning model, offering opportunities for tailored applications based on specific pathologies.
2. Advance beyond conventional evaluation methods by introducing a transparent and clinically consistent framework for validating segmentation models.

By aligning model development with clinical needs, this work seeks to enhance the practical relevance and reliability of deep learning models for brain disease diagnosis and treatment.

The rest of this paper is organized as follows: Section 2 details the materials and methods used: Dataset description, Model description, and evaluation methods. Section 3 presents our results. The discussion and comparison with similar methods are presented in Section 4. In Section 5, we focus on the Limitations and Future perspective of our work. We conclude this paper in Section 6.

## 2. Materials and Methods

The methodology section outlines the dataset used (Section 2.1) and the preprocessing steps (Section 2.2) taken to optimize its use with the U-net model. This is followed by a detailed description of the model itself (Section 2.3)—a U-net model with transfer learning enhanced InceptionV3 as encoder, as well as the hardware and software employed. Lastly, we discuss the evaluation metrics and methods supporting both the overall volume analysis and the single-component analysis approaches (Section 2.4).

### 2.1. Dataset Collection and Preparation

A total of 124 brain MRI examinations with abnormal findings were collected retrospectively between 2021 and 2023 from the 2nd Department of Radiology at Eginition University Hospital in Athens. The examinations were performed using a 3.0T Achieva TX Philips MRI system (Philips Healthcare, Best, The Netherlands), equipped with an eight-channel head coil. The imaging protocol included both conventional and advanced techniques, with T2-weighted (T2), T2 Fluid Attenuated Inversion Recovery (FLAIR), and Gadolinium-enhanced T1-weighted (T1ce) modalities selected for their relevance across the pathologies of interest: tumors, strokes, multiple sclerosis, and white matter hyperintensities (WMH). Experienced MR physicists visually assessed all images for potential artifacts, ensuring quality, while two experienced neuroradiologists provided diagnoses and ground truth labeling using ITK-SNAP (v4.2.0) [23] software by consensus. All diagnoses were cross-evaluated and confirmed using laboratory results and biopsy data to ensure accuracy and reliability of the ground truth annotations. The dataset was divided into a training set of 100 patients, comprising 19,054 2D axial slices, and a validation set of 24 patients, yielding 4320 2D axial slices. The final dataset distribution was achieved by excluding slices that contained artifacts, unrelated pathologies, or significant deformations caused by surgeries, therapeutic interventions, or external factors. Additional exclusion criteria included poor image quality, incomplete scans, misaligned slices, and cases where motion artifacts or improper contrast administration compromised diagnostic clarity.

### 2.2. Image Preprocessing

**Figure 1:**
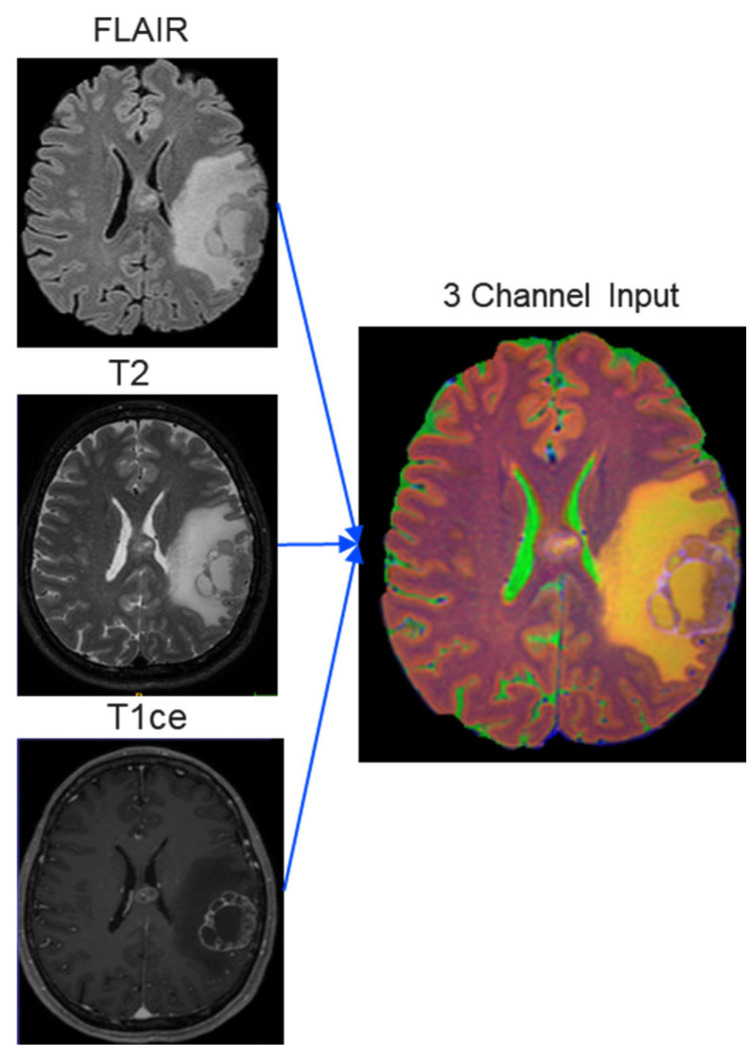
The three MRI modalities registered and combined to act as inputs to U-net model.

Image preprocessing and data augmentation play a vital role in the utilization of CNNs in the medical domain and can efficiently boost performance, decrease data dimensionality, computation complexity, and conversion time [24]. Initially, the original DICOM images were converted to nifty files. Then, the skull was removed, and the axial plane was cropped/filled to 256 × 256 dimensions. T2 and T1ce were registered to FLAIR, and all of them were stretched to the 12-bit intensities range. The ground truths were smoothed to decrease errors during manual labeling. Training images were augmented using horizontal and vertical flips, ±90° range rotations, random zooming from 0% to 20%, shuffled, and presented to the models as new ones. The validation set was not augmented or shuffled. The three MRI modalities were subsequently stacked to act as inputs for the transfer learning model that requires RGB images, as can be depicted in Figure 1.

### 2.3. U-Net Model—Transfer Learning

**Figure 2:**
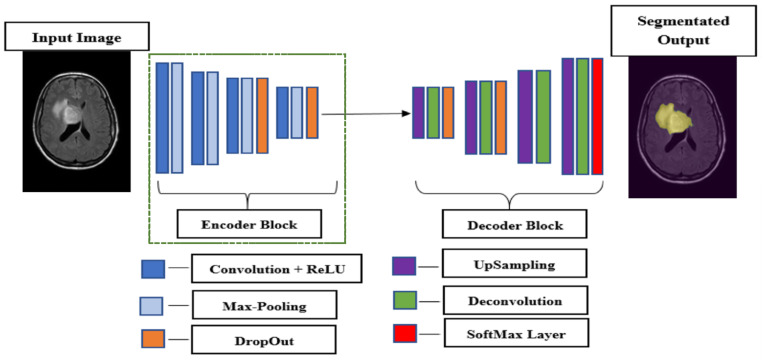
Schematic illustration of U-net architecture with encoder and decoder parts. With green dashed lines is outlined the Encoder part that in our implementation is replaced by the pre-trained InceptionV3 CNN model. The yellow mask on the segmented output image is the segmentation mask produced by our model as the final output.

The U-net architecture is a popular choice for medical image segmentation due to its symmetrical design, comprising two primary components: the encoder and the decoder. The encoder functions as a feature extractor, capturing the contextual information in the input images by progressively reducing their spatial dimensions while increasing the depth of feature maps. This is achieved through successive convolutional and pooling layers [25]. The decoder, on the other hand, aims to reconstruct the segmentation map by up sampling the encoded features back to the original resolution, determining the regions of interest for segmentation. The decoder employs transposed convolutions (or up sampling layers) and concatenates features from corresponding encoder layers through skip connections, which help preserve spatial details and improve segmentation accuracy (Figure 2).

In our implementation, we enhanced the U-net architecture by using InceptionV3 [26] as the encoder, replacing its final layers with custom ones suited for segmentation tasks. The InceptionV3 model, a deep convolutional neural network pre-trained on large-scale datasets, offers several advantages as an encoder. It employs Inception modules, which process the input data using multiple kernel sizes (e.g., 1 × 1, 3 × 3, and 5 × 5 convolutions) simultaneously, capturing features at various scales. Additionally, these modules integrate information from both fine-grained details and abstract patterns, enabling a more robust representation of the input data. By incorporating this powerful encoder into the U-net framework, we leveraged its ability to capture complex spatial and contextual information, thereby enhancing the segmentation performance of our model. In our case, we initiated our model with pre-trained weights from ImageNet of almost 1.2 million natural training images.

Our model was left to train under the hyper-parameters presented in Table 1. For the training, testing, and overall implementation of the models, we used TensorFlow with Keras API [27] and an NVIDIA GPU RTX 4090 24GB (NVIDIA Corporation, Santa Clara, CA, USA) with 32 GB RAM, along with an Intel Xeon E5-1630v3 @3.70 Hz (Intel Corporation, Santa Clara, CA, USA) with 32 GB RAM and Windows 11 (Microsoft Corporation, Redmond, WA, USA) operating system.

### 2.4. Model’s Evaluation Metrics and Methods

#### 2.4.1. Whole Abnormal Volume Evaluation

To evaluate the segmentation performance of our model in a consistent way, we run two validation experiments. On the first one, which is usually met in the literature, the extracted metrics are based on the Whole abnormal volume Evaluation inside an MRI examination. The segmentation metrics were calculated as the average values across the 24 examinations of the validation set after comparing the reference Ground Truths (GTs) with the automated detections of each examination. The selected metrics are the following:

- Dice Similarity Coefficient (*DSC*): The *DSC* measures the similarity between two sets of data, often used to quantify the overlap between detected and ground truth segmentations. It ranges from 0 (no overlap) to 1 (perfect overlap), providing an indication of segmentation accuracy. The formula for *DSC* is:

(1) $$DSC=\frac{2\times \left|A\cap B\right|}{\left|A\right|+\left|B\right|}$$

where *A* is the detected segmentation, *B* is the ground truth and ∣⋅∣ denotes the size of the set.

- *Sensitivity*, or Recall, is the proportion of actual positive cases correctly identified by the model. A high sensitivity indicates a low false negative rate. The formula for sensitivity is:

(2) $$Sensitivity=\frac{TP}{TP+FN}$$

where *TP* is the number of true positives and *FN* is the number of false negatives.

- *Precision*: Precision is the proportion of predicted positive cases that are correctly identified. It reflects the model’s ability to minimize false positives. The formula for precision is:

(3) $$Precision=\frac{TP}{TP+FP}$$

where *TP* is the number of true positives, and *FP* is the number of false positives.

#### 2.4.2. Single Component Abnormal Volume Evaluation

**Figure 3:**
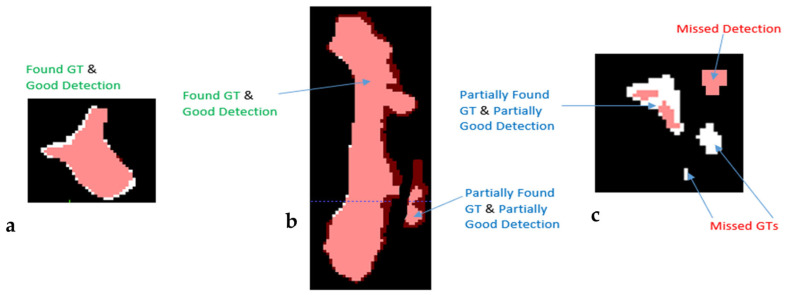
Schematic explanation for terms (**a**) Found GT, Good Detection, (**b**) Partially Found GT, Partially Good Detection, and (**c**) Missed Detection and Missed GT. The detections are marked with red, while the reference GTs are marked white.

On the other hand, the second method presents a deep analysis based on each individual abnormal component (Single Component abnormal volume Evaluation) inside the examinations. The 24 examinations of the validation set contain 867 abnormal GTs components. After discussion with our radiologists and following thresholds align with established practices in segmentation benchmarks [28,29,30], we used the terms reference GT (the ground truth as defined by the experts), Found GT where a reference GT overlaps with enough detection pixels for producing DSC ≥ 0.5 (Figure 3a,b), Partially Found GT for 0.05 ≤ DSC < 0.5 (Figure 3b,c), and Missed GT for DSC < 0.05 (Figure 3c). Respectively, we defined the terms Good Detection, Partially Good Detection, and Missed Detection.

The rationale for selecting “Found”, “Partially Found”, and “Missed” ground truth labels based on Dice Similarity Coefficient thresholds is grounded in practices commonly used in medical imaging and segmentation challenges:

- “Found” (DSC ≥ 0.5): A threshold of 0.5 is frequently used as a minimum for meaningful overlap, as it indicates that at least half of the volume of a segmented region matches the ground truth. This threshold has been applied in tumor segmentation tasks, including challenges like BraTS (Brain Tumor Segmentation) and LiTS (Liver Tumor Segmentation), to identify valid detections where segmentation reasonably approximates the expert annotations [31,32].
- “Partially Found” (0.05 ≤ DSC < 0.5): The range from 0.05 to 0.5 accounts for partial matches where detection may overlap the GT but insufficiently to qualify as a reliable segmentation. This category acknowledges borderline cases that may provide some utility, for instance, hinting at the presence of a lesion but failing in detailed segmentation.
- “Missed” (DSC < 0.05): A DSC below 0.05 indicates negligible overlap, typically attributed to false negatives or detections that do not align meaningfully with the GT. Such cases are considered failed attempts to segment the target correctly.

Finally, the use of these categories and thresholds enables a clear, clinically meaningful evaluation of model performance by distinguishing between successful detections, areas needing improvement, and outright failures. This level of granularity allows for targeted refinements during model development and facilitates robust comparisons across datasets and methodologies, ensuring that the evaluation aligns closely with practical clinical expectations.

#### 2.4.3. Volume, Positional, and Intensity Analysis

We conducted three additional experiments to further evaluate our model’s behavior in relation to the volume, position, and intensity levels of the abnormalities. For volume assessment, we compared the model-generated volumes at both the whole examination level and for individual abnormal components against the corresponding ground truth volumes. For positional analysis, we examined the location of each abnormal component relative to the center of the brain.

**Figure 4:**
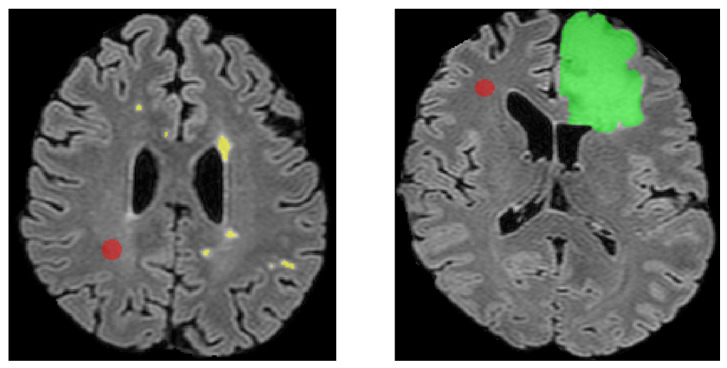
Two different patient examination slices from FLAIR modality from the validation dataset. (**Left**): Whiter matter hyperintensity GT is depicted with yellow color and the Healthy Reference VOI with red. (**Right**): Tumor GT is depicted with green and the Healthy Reference VOI with red.

Lastly, to evaluate the impact of intensity levels across the three modalities, the intensity difference was calculated by comparing the average intensity of each abnormal component with the average intensity of a healthy Volume of Interest (VOI) within the brain’s white matter for each examination (Figure 4). The intensity difference (*ID*) is defined as:

(4) $$ID=\frac{Abnormal\;Intensity_{mean\;}-Healthy\;VOI\;Intensity_{mean}}{Healthy\;VOI\;Intensity_{mean}}\times 100\%$$

where *Abnormal Intensity_mean_* is the mean intensity of the abnormal component and *Healthy VOI Intensity_mean_* is the mean intensity within the healthy VOI in the brain’s white matter.

We utilized the Python libraries Pandas [33] and Matplotlib [34] for extracting and visualizing our results.

## 3. Results

### 3.1. Whole Abnormal Volume Evaluation

The segmentation metrics were computed as the average values across the 24 validation set examinations by comparing the automated detections with the reference ground truths. Metric results and their statistics, namely, DSC, Sensitivity, and Precision, are presented in Table 2.

The mean values of DSC, Sensitivity, and Precision are 0.76, 0.78, and 0.82, respectively.

**Figure 5:**
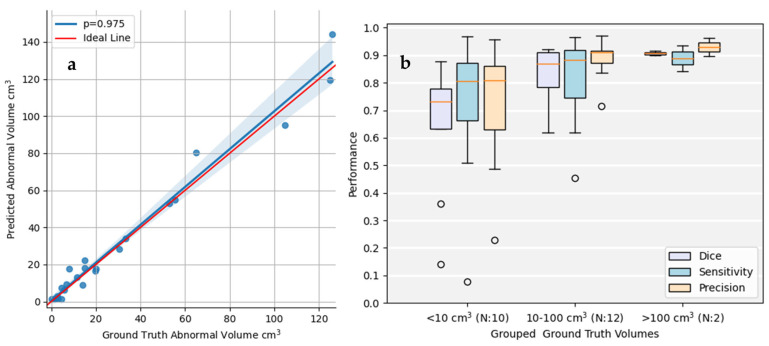
(**a**) Graphic illustration of the correlations among Ground Truth Volumes (cm^3^) and Detected abnormal Volumes (cm^3^). Each bullet represents a patient MRI exam from the validation set. (**b**) Grouped analysis for the examinations that contain less than 10 cm^3^, 10 cm^3^–100 cm^3^, and bigger than 100 cm^3^ of abnormal volume. The orange line is the mean value and the white circles are the outliers.

In Figure 5a, where the detected and reference GTs volumes are compared, it can be observed that volume-wise the model produces similar detections to the ground truths, with a high correlation of ρ = 0.975. The quality of the detections can be further investigated by grouping the validation set by volume into three subsets as depicted in Figure 5b. Our model, for the examinations that contain abnormal volumes higher than 10 cm^3^, produces metric numbers close to 0.9. However, for smaller volumes, there is a 10–15% performance drop.

**Figure 6:**
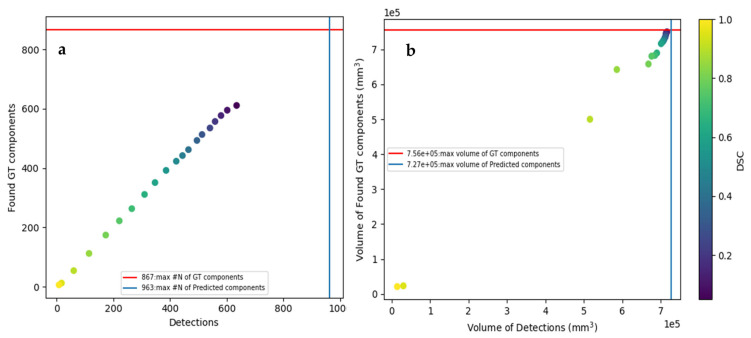
Graphical illustration of the (**a**) total Found GT components versus the Detected Components; (**b**) Found GTs Volume versus the Detections’ Volume for different Dice Coef. Thresholds.

Further investigation of different DSC thresholds—for 0.05 to 0.95 DSC with a step of 0.05—(Figure 6a) reveals an almost linear correlation between the Found GT components and the Good Detections number. It is worth mentioning that in the 0.05–0.5 threshold range, the distance between points is doubled compared to the 0.5–1.0 threshold range. This leads to the indication that the model presents a higher detection rate at low DSC levels compared to the higher ones. On the other hand, on the relevant volume graph (Figure 6b), almost 85% of the GT volume is promptly detected at DSC levels as high as that of 0.80, highlighting the strong influence of the biggest components.

### 3.2. Single Component Volume Evaluation

The 24 examinations of the validation set contain 867 abnormal GTs components, from which the model found correct 423, partially good 235, and missed 209 of them. Subsequently, of the 963 produced components as detections, 422 were good, 299 were partially good, and 242 were missed with little to no overlap with ground truth (Table 3).

As can be depicted in Table 3, the Found GTs and the Missed GTs count for 48.8% and 24.1% of the total GT components and for 95.6% and 0.5% of the total GT volume, respectively. Similarly, the Missed Detections account for 25.1% of the detections and for 1.0% of the detected volume. As expected, there is a clear pattern pointing out that the model can detect bigger components and thus most of the total abnormal volume.

### 3.3. Single Component Position Evaluation

**Figure 7:**
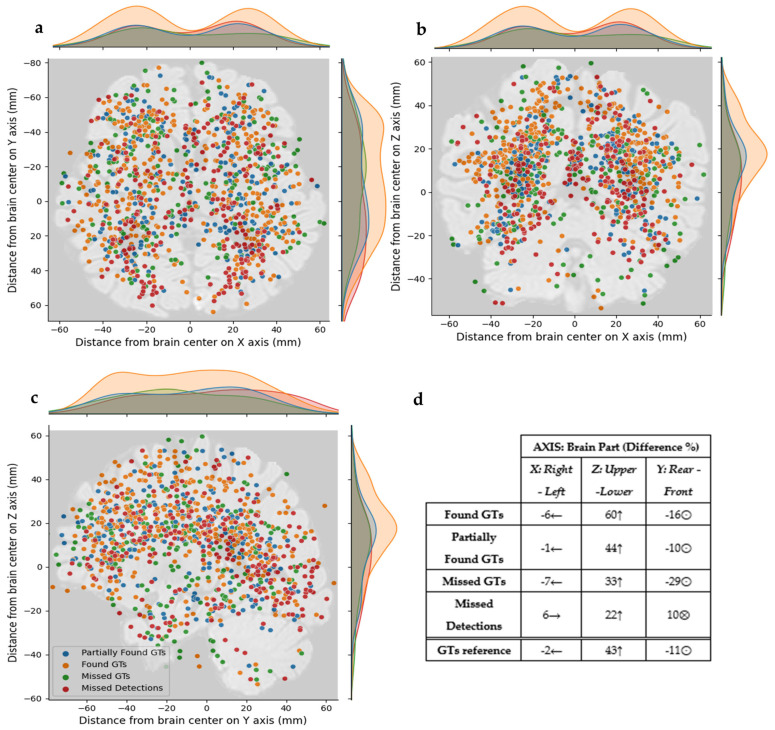
Distribution of the abnormal GT components and abnormal detections for (**a**) the axial plane (X-Y), (**b**) the coronal plane (X-Z), and (**c**) the sagittal plane (Y-Z). (**d**) The percentage difference of the concentration of detections and GTs for the X, Y, and Z axes and their relevant positions around the brain center. For anatomical reference the ←, →, ↑, ⨀, ⨂ stand for right, left, upper, front and rear parts of the brain.

Concerning the positional analysis, the distribution of the center of mass for the abnormal components across the three axes is depicted in Figure 6, and it reveals patterns for the model’s behavior with respect to the components’ positions inside the brain. As can be observed in the density graphs of Figure 7a–c, the Found GT components are mostly located ±20 mm, among +30 mm and −40 mm, and around +20 mm distance from the brain center at the X, Y, and Z axes, respectively. Similarly, the Partially Found GTs are located around the ±20 mm points on the X axis, mostly around the +20 mm point on the Y axis, and around the 20 mm point on the Z axis. The Missed GTs are concentrated mostly around −20 mm points on X and Y axes and around 20 mm points on Z axis. On the contrary, the Missed Detections are mostly located around +20 mm points on the X and Y axes and around 0 mm points on the Z axis. As can be noticed in Figure 7d, the concentrations of the GTs and detections are mostly found on the left, on the upper, and on the rear part of the brain, with the exception of the Missed Detections that are observed on the left, on the upper, and on the front part of the brain. In more detail, there are 6% more Missed Detections on the left part of the brain compared to the right part, 22% more on the upper part against the lower part, and 10% more on the front part compared to the rear.

### 3.4. Single Component Intensity Evaluation

**Figure 8:**
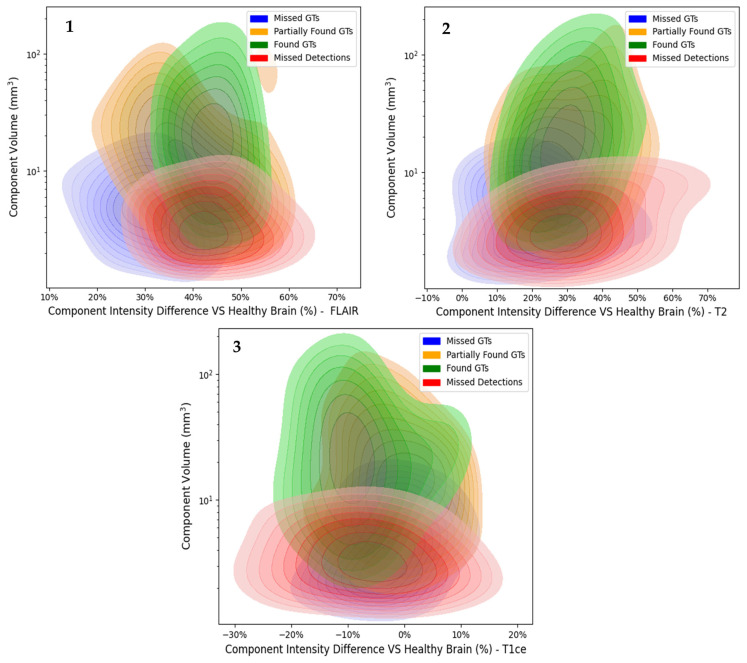
KDE plots of the Abnormal Component Volumes versus their intensity difference compared to the healthy reference VOI for (**1**) FLAIR (**2**) T2 (**3**) T1ce modalities.

The influence of the intensity levels against the single abnormal components’ volume for the three MRI modalities is described in Figure 8. The model is able to produce Found GTs of almost every volume, Partially Found GTs up to 2 × 10^2^ mm^3^, Missed GTs smaller than 200 mm^3^, and Missed Predictions up to 100 mm^3^. According to Figure 7, the intensity difference of the majority of the Found GTs has a range of approximately 30% to 55% for FLAIR, 10% to 50% for T2, and −20% to 10% for T1ce. The Partially Found GTs present a range of 20% to 60% for FLAIR, 10% to 55% for T2, and −20% to 15% for T1ce. Similarly, Missed GTs are located in the intensity range of 10% to 45% for FLAIR, 0% to 50% for T2, and −20% to 10% for T1ce. Finally, the model Detections of 25% to 65%, 0% to 70%, and −30% to 20% intensity range for FLAIR, T2, and T1ce, respectively. The complete distribution for all the single components can be found in Supplementary Figure S1. 

## 4. Discussion

In this study, we aimed to evaluate the performance of a pre-trained U-net segmentation model on multi-pathology MRI examinations, focusing on its ability to identify and characterize abnormalities based on volume, spatial position, and intensity. The analysis included assessing the model’s accuracy in segmenting both overall abnormal volumes and individual pathological components within the dataset. This work also introduces a novel and systematic framework for evaluating segmentation models in clinically diverse scenarios.

### 4.1. Whole Abnormal Volume Evaluation Assessment

Comparing our results with established methods (Table 4) from previous research demonstrates consistency in the model’s performance characteristics. The mean DSC of 0.76 aligns well with expectations derived from analogous studies, reaffirming the reliability and robustness of the U-net architecture in segmenting abnormal structures across diverse datasets and experimental setups. Comparing further our model with the previous studies, we noticed the similarities in segmenting large and medium size abnormalities and the performance drop when it comes to small size volumes.

Delving deeper into Table 4, it is essential to consider the clinical context of each dataset and pathology. Tumors are typically larger in size, strokes can vary widely in dimensions, while MS lesions and WMHs are generally smaller and diffusely distributed within the brain. These differences account for the varying performance levels observed across different models, particularly in cases involving pathological volumes of diverse sizes. This underscores the importance of factoring in lesion-specific characteristics when evaluating segmentation algorithms [35,36,37].

**Figure 9:**
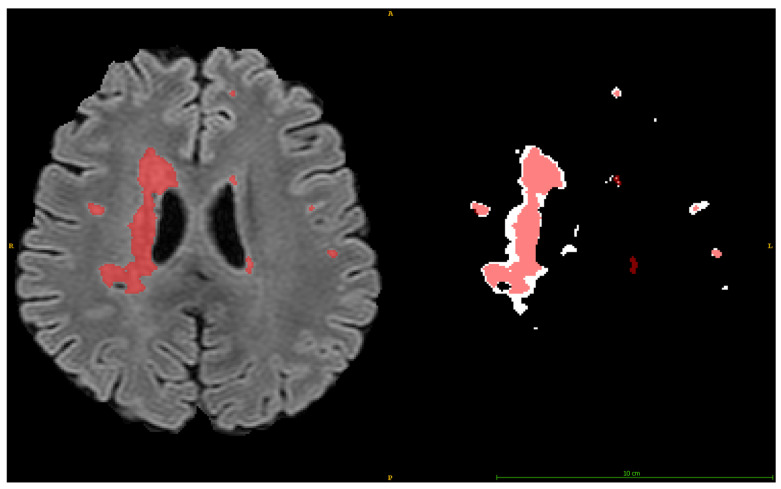
White matter hyperintensity patient examination slice from the validation dataset. The Produced Detection is depicted in red, while the Ground Truth (GT) is depicted in white.

Visual inspection of example slices from examinations exhibit varying performance levels. Figure 9, a randomly selected patient examination from the entirety of the validation dataset, offers additional insights into our model’s segmentation quality. More precisely, the examination’s DSC equals 0.85. Despite the high DSC, it can be clearly depicted that micro-abnormalities of great importance are either missed or under-segmented.

### 4.2. Single Component Abnormal Volume Evaluation Assessment

The observations and results of the strengths and limitations of the U-net model’s performance led to the necessity of further analysis in the field of Single-Component Abnormal Volume.

The results of Table 3 showed that 99% of the total abnormal volumes were successfully detected. Thus, this 1% of the volume that was lost due to missed detections accounts for nearly 200 micro abnormalities, a number that holds immense clinical importance. In clinical practice, micro strokes and early-stage tumors often manifest as abnormalities of very small sizes, posing challenges in their detection and diagnosis. For instance, in the case of strokes or micro ischemic WHM, the size can vary but is often characterized by affecting a small volume of brain tissue, generally less than 15 milliliters in volume [38]. Furthermore, regarding early-stage brain tumors, their size may range from a few millimeters to a few centimeters in diameter [39]. Moreover, some tumors maybe even smaller, measuring only a few millimeters across [40]. These tumors are often detected incidentally or during routine imaging studies such as MRI or CT scans. Our model missed 25% of the GT abnormalities and produced 25% empty detections. This underscores the importance of focusing on improving the model’s performance in detecting smaller abnormalities, as these can have significant clinical implications for patient survival rate and treatment planning.

### 4.3. Positional Analysis Assessment

It is important to note that the size of a brain abnormality alone does not necessarily indicate its severity or aggressiveness [41]. Other factors, such as the location within the brain, are also critical considerations for accurate and early detection [42]. Indeed, our results indicated that the location of abnormalities within the brain plays a pivotal role in the model’s performance in detection. Specifically, abnormalities situated in certain regions, such as the deep white matter or around ventricles, were more conducive to accurate detection by the model. This is evident in the positional analysis of Figure 6, where concentrations of Found GTs or Partially Found GTs components are observed in the aforementioned areas within the brain. Conversely, in Figure 6, regions such as the cerebellum, gray matter, or cortex boundaries presented more prevalent missed detections. The observed patterns suggest that the model’s performance may be influenced by factors such as tissue density, structural complexity, and proximity to anatomical landmarks. Abnormalities located in regions with relatively uniform tissue characteristics, such as the deep white matter, may present clearer contrasts against surrounding tissue, facilitating their detection by the model. Furthermore, the results of Figure 6 attest to the conclusion that Found or Partially Found GTs follow the reference GTs distribution with less than 5% differences in all axes, while the Missed Detections behave conversely and appear mostly in the rear and right part of the brain. Our model is missing abnormalities mostly in the left hemisphere and closer to the center-rear part of the brain. Thus, this is arising an immense need for creating datasets with reference GTs with equal distribution in the brain and models that could segment homogeneously over the brain.

### 4.4. Intensity Analysis Assessment

The findings reveal distinct patterns in the intensity differences of Found GTs, Partially Found GTs, Missed GTs, and Missed Detections across FLAIR, T2, and T1ce modalities, as can be depicted in Figure 7. Notably, Found GTs exhibit intensity differences within specific ranges for each modality characteristic of each abnormality type.

Notably, Found GTs tend to exhibit intensity differences within high contrast ranges for each modality, characteristic of each abnormality type [43]. For example, tumors or tumors like Multiple Sclerosis typically present high contrast in the entirety of our three modalities. Of particular interest is the observation that Missed GTs are often located in FLAIR areas where the intensity difference with healthy tissue is low or in T1ce areas where the intensity difference with healthy tissue is high. Furthermore, the intensity ranges of Missed Detections extend to negative values, particularly in T2 and T1ce modalities. This phenomenon suggests that hypointense regions may be more difficult to detect by the model, probably because they deviate from typical intensity patterns observed in healthy tissue. This underscores the model’s capacity to identify abnormalities based on intensity variations, albeit with limitations in certain modalities and regions.

## 5. Limitations—Future Perspectives

While our model demonstrated high performance on this clinical custom dataset, it still exhibits limitations in detecting smaller abnormalities. To address this, we plan to expand our dataset by including a broader range of small-sized pathologies, incorporating advanced imaging modalities, such as DTI or PET, to complement standard MRI sequences, and to add, both for training and validation, publicly available datasets. Additionally, we aim to enhance our model by incorporating additional layers and specialized functions to improve its performance, particularly in the outer regions of the brain and for abnormalities with larger intensity variations.

Furthermore, we intend to conduct a more in-depth analysis of our model’s behavior by generating detailed detection and segmentation results for each pathology separately. This will allow us to develop more specialized and tailored tools for clinicians, enhancing the model’s utility in clinical practice.

## 6. Conclusions

In conclusion, our study comprehensively analyzed the performance of a pre-trained U-net segmentation model on multi-pathology MRI examinations, with a specific focus on identifying patterns related to abnormal component volume, position, and intensity. Through meticulous evaluation using both the common evaluation metrics and focusing on each abnormal component, we highlighted both the strengths and weaknesses of the model, offering valuable insights for its application in clinical settings. As bottom line, we suggest that in addition to the commonly reported statistical metrics such as DSC score, sensitivity, and precision, researchers should also provide detailed information on the exact number and volume of detected and missed components, their spatial distribution within the brain, and their behavior relative to the intensity levels of the utilized modalities. Incorporating these factors will align AI models more closely with clinical intuition, facilitating their seamless integration into routine medical practice.

## Figures and Tables

**Table 1:** U-net model training hyper-parameters.

Image size	256 × 256 × 3
Training Epochs	100
Batch Size	32
Loss Function	Dice loss + Cross-Entropy loss
Not Optimizer	Adam
Learning Rate	0.00001
Model selection Criteria	Best validation DSC

**Table 2:** DSC, Sensitivity, and Precision results and statistics.

	DSC	Sensitivity	Precision
Mean	0.76	0.78	0.82
STD	0.18	0.20	0.16
Median	0.80	0.85	0.88
25 Quant.	0.74	0.75	0.82
75 Quant.	0.89	0.91	0.91
Min.	0.14	0.08	0.23
Max.	0.92	0.97	0.97

**Table 3:** (**Top**) Component base results regarding the Found Ground Truths, the Partially found Ground Truths, and the Missed Ground Truths for the 24 examinations of the validation set; (**Bottom**) Component base results regarding the Good Detections, the Partially Good Detections, and the Missed Detections for the 24 examinations of the validation set.

Found GTs (True Positives)			Partially Found GTs			Missed GTs (False Negatives)			Total GTs
DSC ≥ 0.5			0.05 ≤ DSC < 0.5			DSC < 0.05			
#N	% GTs	% GT Volume	#N	% GTs	% GT Volume	#N	% GTs	% GT volume	
423	48.8	95.6	235	25.1	3.9	209	24.1	0.5	867
Good Detections			Partially Good Detections			Missed Detections (False Positives)			Total Detections
DSC ≥ 0.5			0.05 ≤ DSC < 0.5			DSC < 0.05			
#N	% Detections	% Detected Volume	#N	% Detections	% Detected Volume	#N	% Detections	% Detected Volume	
422	43.9	97.2	299	31.0	1.8	242	25.1	1.0	963

**Table 4:** Detailed comparison table between previous studies with similar characteristics. * SD: Small Volume Detections; MD: Medium Size Detections; LD: Large Size Detections. Categories are following the limits of Figure 5b.

	Model	Pathology	Modalities	DSC Score			
Zhang et al. [15]	Preprocessing methods + U-net	Tumors	T1, T2, FLAIR, T1ce	0.87			
Gab Allah et al. [16]	Edge-U-net	Tumors	T1ce	0.88			
Ferreira et al. [17]	U-net + Data Augmentation with GANs	Tumors	T1, T2, FLAIR, T1ce	0.90			
Huang et al. [18]	Transfer learning + CNN	MS	FLAIR, T1ce	0.66			
Pooya Ashtari et al. [19]	Pre-U- net	MS	FLAIR	0.4			
Yunzhe et al. [20]	Multi-path CNN	Strokes	T1, FLAIR	0.5			
Wu et al. [21]	SC U-net	WMHs	T1, FLAIR	0.78			
Guerrero et al. [22]	uResNet	Strokes, WMHs	T1, FLAIR	0.4–0.69			
Our method	Transfer learning + InceptionV3 encoder + U-net	Strokes, WMHs, MS, Tumors	T2, FLAIR, T1ce	Mean: 0.76	SD *: 0.74	MD *: 0.87	LD *: 0.91

## Data Availability

All data generated or analyzed are available from the corresponding author upon reasonable request.

## Supplementary Materials

The following supporting information can be downloaded at: https://www.mdpi.com/article/10.3390/jimaging11010006/s1, Supplementary Figure S1: Scatter plots of the Abnormal Component Volumes versus their intensity difference compared to the healthy reference for (1) FLAIR (2) T2 (3) T1ce modalities.
