# Supplementary material for: Exploring Multi-Pathology Brain Segmentation: From Volume-Based to Component-Based Deep Learning Analysis
Source: J Imaging. 2024 Dec 31;11(1):6. doi: 10.3390/jimaging11010006 (PMC11766070; doi:10.3390/jimaging11010006)
Supplement: Supplementary file 1 [file jimaging-11-00006-s001.zip › jimaging-3390610-supplementary.pdf]

Supplementary File S1

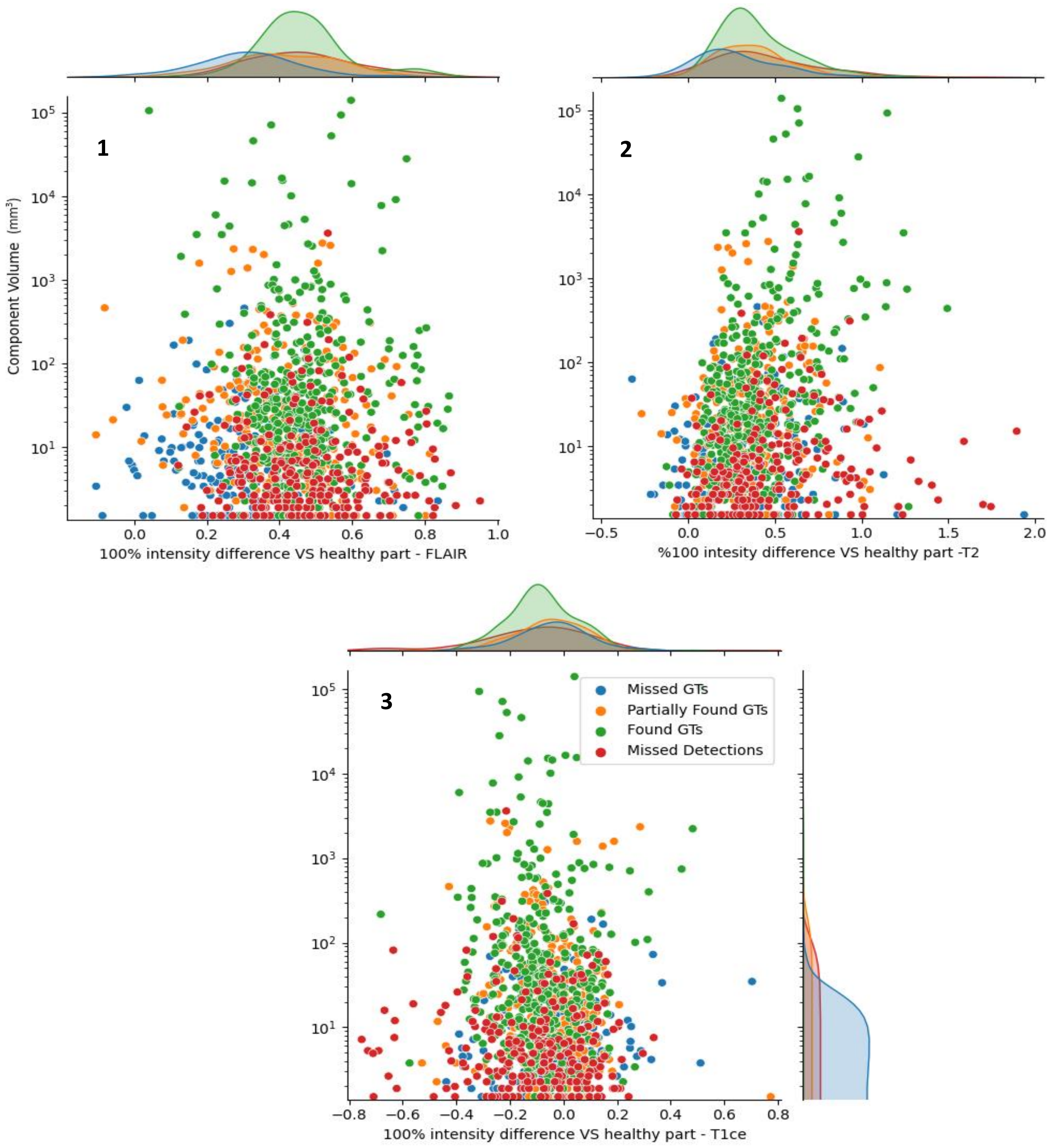

Supplementary Figure S1: Scatter plots of the Abnormal Component Volumes versus their intensity difference compared to the healthy reference for 1) FLAIR 2) T2 3) T1ce modalities.
